# Danish Medical Institutions

**Published:** 1903-10-17

**Authors:** 


### Editor's Letter-Box.

**[Our Correspondents are reminded that prolixity is a great bar to publication, and that brevity of style and conciseness of statement greatly facilitate early insertion.]**

### DANISH MEDICAL INSTITUTIONS.

Dr. George Graham writes: "Tak for mad" not ("maal") can hardly be said for the literary meal your correspondent has given us in writing of "Finsen's Institute" under "Danish Medical Institutions" in your issue of October 3rd, as many parts of it are very indigestible. A slight computation would have shown that with the lamps described 34 patients could not be treated at one time. As a matter of fact there are seven large 4-light lamps in the chief ward.

The French lamp—by which is meant, I presume, the Lortet-Genoud and not the Broca-Chatin—is thoroughly well known by the head sister, Miss Forman, and her chief assistants, and your correspondent is evidently not aware, probably owing to her hurried visit, that the medical authorities there have tried it fully in the laboratory—as they do nearly every new lamp that comes out—and discarded it. Its use has also practically been given up at the St. Louis Hospital in Paris.

Contrary to the description, it is the Finsen lamp that has all the advantages in the variety of the compressing lenses (made of quartz, not glass as stated) and in the treatment of corners and curves in the face, and its superiority as regards the actual results of treatment is unquestioned.

The short exposure given with the Lortet-Genoud of 20 minutes, instead of the hour with the Finsen, may be a great saving of time, but it is playing at the treatment for the patient. It seems to me, therefore, that your correspondent's suggestion that both sorts of lamps should be found in the institute is more than a bold one, and might also prove very misleading to some of your readers.

"Lounging in deck chairs and sometimes drinking milk" may be one cause for the patients improving more rapidly in Copenhagen than when treated in some of the London hospitals, but it can only be a very trifling cause, and that it is very far from being the chief one is well known to those who have experienced the treatment in both places. The fees charged in Copenhagen used to be 100 kr. for every month of 26 sittings, but they have recently been raised and approximate more nearly those charged at the London Hospital.

*** Our correspondent was familiar with the light treatment in London before visiting the Danish institution, and was well able to form a judgment as to the results following the use of the French lamp. The error in the number of lamps escaped notice owing to the writer being absent and without the original notes. There is no error in the statement that a patient was paying 100 kr. per month when the article was written. If higher fees prevail now an alteration has been made. Our correspondent is prepared to maintain that the healthy environments at the Copenhagen institution are very conducive to a cure. — Ed. The Hospital.

